# Arginine Induced *Streptococcus gordonii* Biofilm Detachment Using a Novel Rotating-Disc Rheometry Method

**DOI:** 10.3389/fcimb.2021.784388

**Published:** 2021-11-05

**Authors:** Erin S. Gloag, Daniel J. Wozniak, Kevin L. Wolf, James G. Masters, Carlo Amorin Daep, Paul Stoodley

**Affiliations:** ^1^ Department of Microbial Infection and Immunity, The Ohio State University, Columbus, OH, United States; ^2^ Department of Microbiology, The Ohio State University, Columbus, OH, United States; ^3^ Department of Mechanical and Aerospace Engineering, The Ohio State University, Columbus, OH, United States; ^4^ Colgate-Palmolive Technology Center, Piscataway, NJ, United States; ^5^ Department of Orthopedics, The Ohio State University, Columbus, OH, United States; ^6^ National Biofilm Innovation Centre (NBIC) and National Centre for Advanced Tribology at Southampton (nCATS), University of Southampton, Southampton, United Kingdom

**Keywords:** viscoelasticity, biophysical, mechanics, *Streptococcus gordonii*, arginine, dental plaque

## Abstract

Oral diseases are one of the most common pathologies affecting human health. These diseases are typically associated with dental plaque-biofilms, through either build-up of the biofilm or dysbiosis of the microbial community. Arginine can disrupt dental plaque-biofilms, and maintain plaque homeostasis, making it an ideal therapeutic to combat the development of oral disease. Despite our understanding of the actions of arginine towards dental plaque-biofilms, it is still unclear how or if arginine effects the mechanical integrity of the dental plaque-biofilm. Here we adapted a rotating-disc rheometry assay, a method used to quantify marine biofilm fouling, to study how arginine treatment of *Streptococcus gordonii* biofilms influences biofilm detachment from surfaces. We demonstrate that the assay is highly sensitive at quantifying the presence of biofilm and the detachment or rearrangement of the biofilm structure as a function of shear stress. We demonstrate that arginine treatment leads to earlier detachment of the biofilm, indicating that arginine treatment weakens the biofilm, making it more susceptible to removal by shear stresses. Finally, we demonstrate that the biofilm disrupting affect is specific to arginine, and not a general property of amino acids, as *S. gordonii* biofilms treated with either glycine or lysine had mechanical properties similar to untreated biofilms. Our results add to the understanding that arginine targets biofilms by multifaceted mechanisms, both metabolic and physical, further promoting the potential of arginine as an active compound in dentifrices to maintain oral health.

## Introduction

Biofilms are communities of microorganisms, encased in an extracellular polymeric slime (EPS). These communities adhere at either surface interfaces or to neighboring microorganisms (Bjarnsholt et al., 2013). Biofilms are responsible for a number of infectious diseases, where these communities are highly recalcitrant to traditional therapies, promoting the persistence of these infections (Hall-Stoodley et al., 2004). Dental plaque is perhaps one of the most widely understood biofilms affecting human health. Oral pathologies typically arise due to poor oral hygiene and diet, that lead to dental plaque build-up or dysbiosis of the plaque microbial community. Together these factors can lead to oral diseases including dental caries, gingivitis and periodontitis (Mosaddad et al., 2019). Oral hygiene, including combinations of mechanical dental plaque removal and antimicrobial agents in dentifrices, continues to be the most effective method at preventing the development of these pathologies.

Exogenous arginine has emerged as a novel therapy to combat dental plaque. This mechanism has been chiefly attributed to the buffering capacity of arginine metabolism by arginolytic organisms, including *Streptococcus gordonii*. These organisms encode an arginine deiminase system (ADS), which metabolizes arginine, producing ammonia (Wijeyeweera and Kleinberg, 1989a; Wijeyeweera and Kleinberg, 1989b; Jakubovics et al., 2015). This in turn neutralizes acid produced by acidogenic organisms, maintaining a neutral pH within the dental plaque-biofilm (Wijeyeweera and Kleinberg, 1989a; Wijeyeweera and Kleinberg, 1989b). Exogenous arginine treatment also promotes *S. gordonii* growth and prevents the out-growth of cariogenic species, including *Streptococcus mutans*, in mixed species biofilm models (He et al., 2016; Bijle et al., 2019).

Exogenous arginine treatment can also reduce microbial coaggregation (Ellen et al., 1992; Kamaguch et al., 2001; Levesque et al., 2003), and alters the EPS biochemical composition, by preventing the out-growth of *S. mutans*, and subsequently reducing the amount of insoluble glycans produced by this organism (Kolderman et al., 2015; He et al., 2016). Interestingly, treatment with low concentrations of arginine promotes the growth of *S. gordonii* biofilms, however, high concentrations of the amino acid reduces biofilm biomass (Jakubovics et al., 2015). It was predicted that arginine treatment inhibited cell-cell interactions within the biofilm (Jakubovics et al., 2015). Taken together these data suggest that exogenous arginine treatment can disrupt dental plaque-biofilm, preventing its build-up (Kolderman et al., 2015; Manus et al., 2018; Wolff and Schenkel, 2018).

Despite the above observations, there is little understanding of how arginine treatment impacts the mechanical integrity of dental plaque-biofilms, an important factor in understanding how antimicrobials may penetrate the biofilm or how mechanical disruption may physically remove the biofilm. Atomic force microscopy (AFM) showed that *S. mutans* biofilms, grown in the presence of arginine, had reduced adhesion forces to the AFM tip (Sharma et al., 2014). This was predicted to be due to reduced glycan production or hydrogen bonds within the EPS (Sharma et al., 2014). However, effects of arginine treatment on the bulk biofilm properties and biofilm removal have yet to be considered. Furthermore, most studies have focused on how arginine impacts *S. mutans* biofilms, or caries-active plaque (Wolff and Schenkel, 2018). Few have focused on understanding how arginine impacts non-cariogenic plaque, or the biofilms of early plaque colonizers, such as *S. gordonii* (Jakubovics et al., 2015).

Rotating discs have long been used to analyze how biofilm fouling effects the hydrodynamics and drag associated with marine biofouling (Granville, 1982). The disc is rotated at increasing angular velocity, and the resulting torque (resistance to imparted rotary motion) is measured. Increases in torque is related to biomass, roughness and deformability of the biofilm (Dennington et al., 2015; Dennington et al., 2021). Conventionally, such discs are large [i.e. between 0.2 - 1 m diameter (Nelka, 1973; Schultz and Myers, 2003)], and hence cumbersome to manage. However, recently non-contact rotating-disc rheometry has been used to analyze drag associated with marine biofouling on discs 2.5 - 4 cm in diameter (Dennington et al., 2015; Dennington et al., 2021). In this method a rheometer is used as a highly sensitive torque monitor, allowing precise measurements of torque, even that generated by small discs compatible with the scale of routine laboratory biofilm growth systems (Dennington et al., 2015; Dennington et al., 2021). As such, it represents a novel method for direct quantification of biofilms outside of traditional assays, such as microscopic examination, viable counts and crystal violet staining. In addition, it allows real time correlation between imposed shear stress and changes in torque when biofilm is detached, informing how much shear is required to disrupt the biofilm. Here we adapted rotating-disc rheometry to study *S. gordonii* biofilm detachment after arginine treatment.

## Results

### Adapted Rotating-Disc Rheometry Is Sensitive at Detecting Biofilm Rearrangement and Detachment Events

Mechanical analysis of biofilms is becoming more widespread in the field (Gloag et al., 2019). However, analyses of biofilm mechanics in the context of biofilm removal is currently lacking in the field. To meet this need we adapted rotating-disc rheology to analyze biofilm detachment from surfaces.

**Figure 1:**
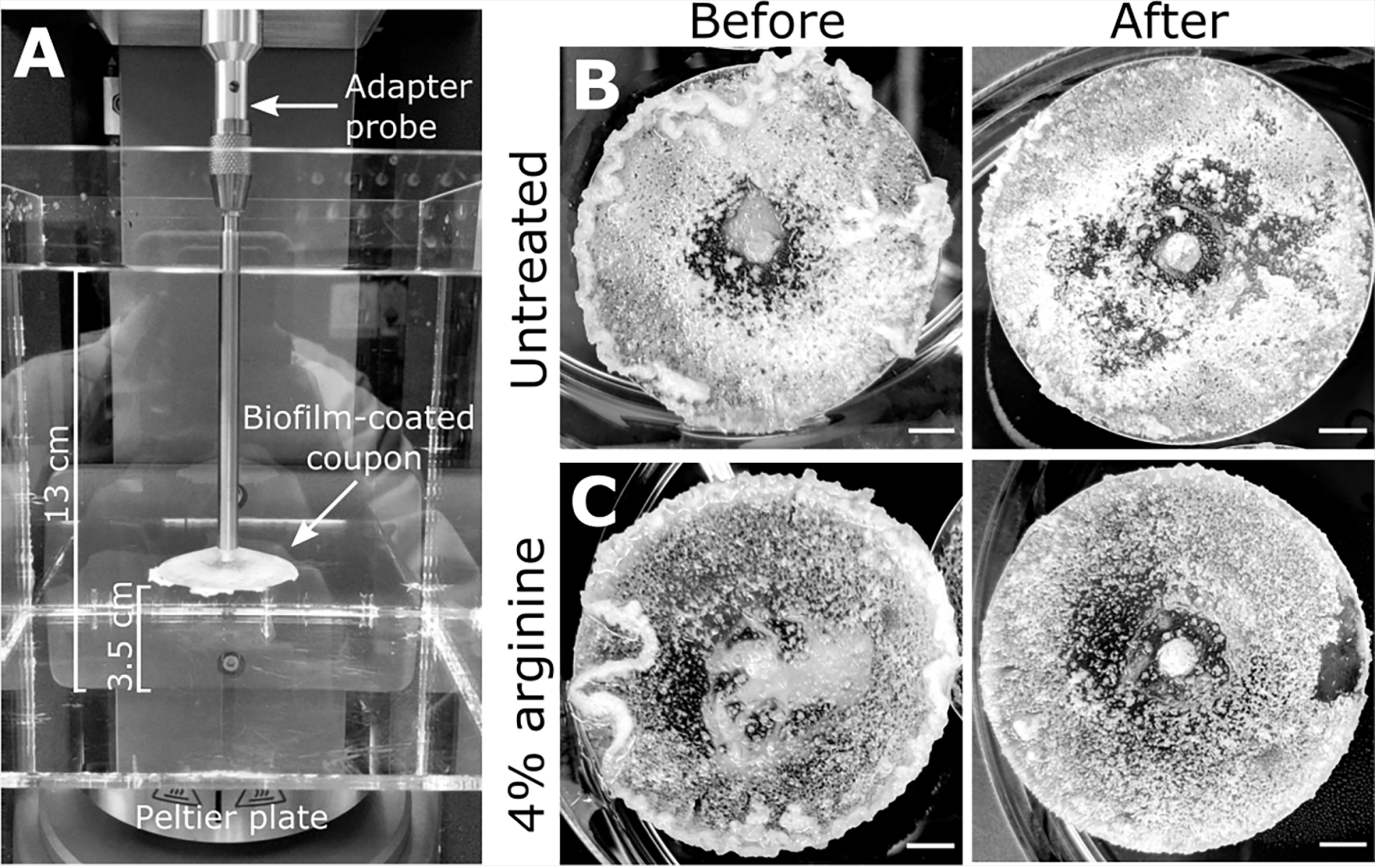
*S. gordonii* biofilms before and after analysis. **(A)** Experimental design for the adapted rotating-disc rheometry analysis. Biofilm-coated coupons were attached to an adaptor probe on the rheometer using a threaded tap that was printed onto the back of the coupon. This was immersed in a container filled with reverse osmosis water. A gap thickness of 3.5 cm was set between the coupon and the bottom of the container. Prior to analysis 7 day *S. gordonii* biofilms, grown on the coupons, were treated with either **(B)** PBS (untreated control) or with **(C)** 4% arginine (labeled). Images depict biofilms before and after rheometry analysis (labeled). Scale bar indicates 5mm.

**Figure 2:**
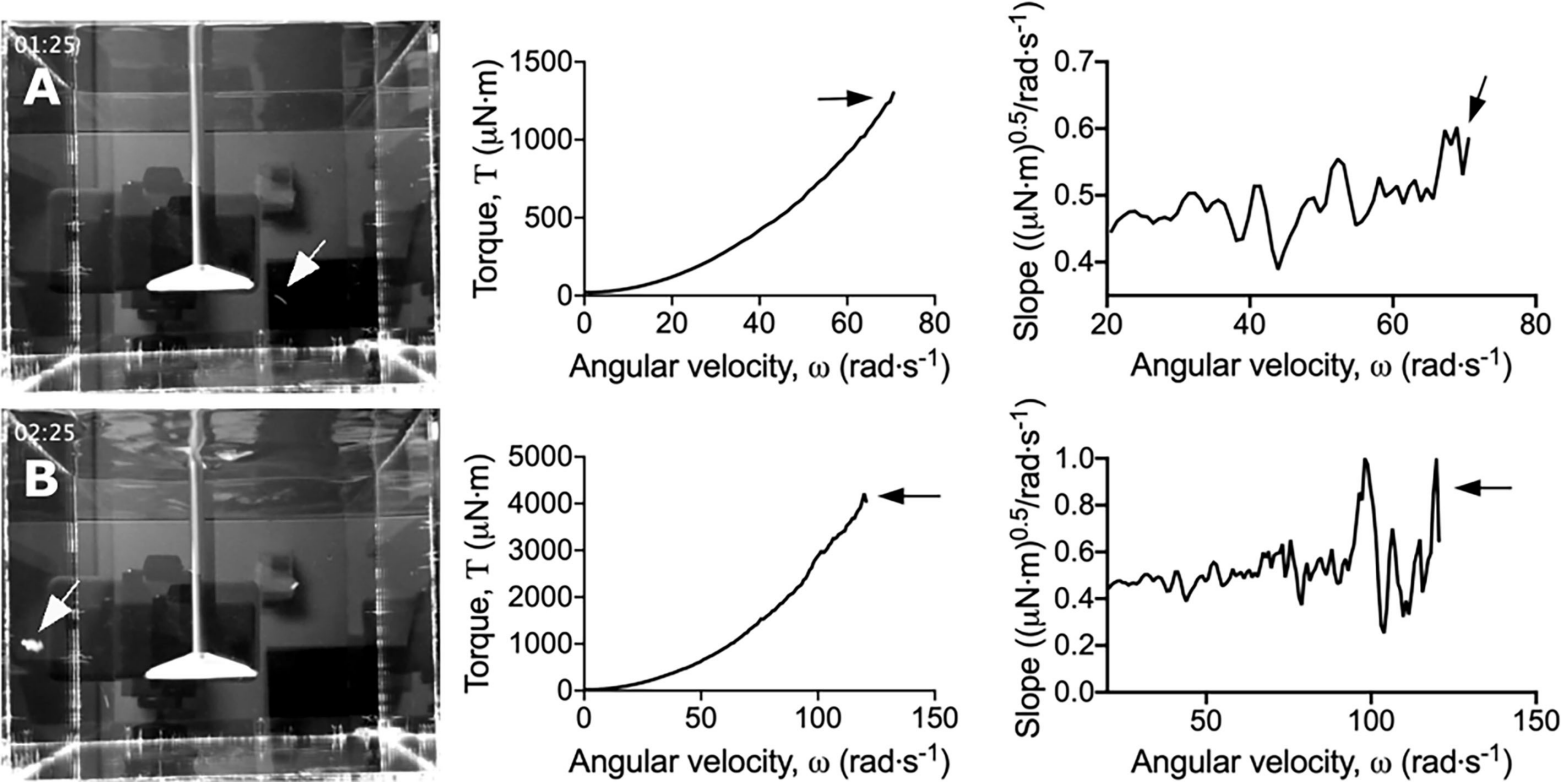
Dips in the torque – angular velocity curve correlate to biofilm detachment events from the coupon. Stills taken from **Movies S1** and **S2** (left panel) depicting **(A)** small and **(B)** large biofilm detachment events. The torque – angular velocity curve (middle panel) and transformed linearized analysis (right panel) at each time is depicted. White arrows indicate detached biofilm and black arrows indicate the corresponding changes in the curve.

*S. gordonii* biofilms were grown on 3D printed coupons for 7 days. Biofilm coated coupons were connected to the rheometer and immersed in reverse osmosis water (**Figure 1A**). Coupons were spun across an angular velocity range of 0.1 – 300 rad·s^-1^ over 360 s, and the resulting torque, a measurement of resistance to rotation, was measured (**Movie S1**; https://doi.org/10.5061/dryad.p8cz8w9q2). Across this velocity range, detachment of biofilm aggregates was observed, particularly at the higher velocity regimes. These detachment events appeared to correlate to reductions in torque (**Movie S1**), with both small (**Figure 2A**) and larger (**Figure 2B**) aggregate detachments detected. After analysis there remained biofilm still attached to the coupon surface (**Figure 1B**). The remaining biofilm was not removed with repeated analysis (**Figure S1**).

To more easily observe the changes in torque associated with biofilm detachment, the torque – angular velocity data was first linearized and then transformed by determining the running slope of 5 consecutive data points (**Figure S2**). Using this transformed analysis, the reductions in torque were emphasized by being visualized as large peaks (**Figure 2** and **Movie S2**; https://doi.org/10.5061/dryad.p8cz8w9q2). Furthermore, changes in torque not associated with macroscopic aggregate detachment were observed, particularly at the lower velocity regimes (**Movie S2**). This suggested that the adapted rotating-disc rheometry analysis was capable of detecting microscopic detachment events, or rearrangement of the biofilm structure in response to external shear stress.

### Arginine-Treated Biofilms Are More Sensitive to Removal by Shear Stresses

Having validated the sensitivity of the adapted rotating-disc rheometry, we used this assay to determine how arginine treatment influenced biofilm mechanics, in regards to biofilm removal. Seven day *S. gordonii* biofilms were treated with either PBS (untreated control), 4% arginine, or equal molar concentrations of glycine or lysine (0.23M) for 2 min. This short treatment time was selected to mimic the time that a person would typically carry out their routine oral hygiene regimen. Glycine and lysine were selected as control amino acids, to determine if any biofilm disrupting effects were a general property of amino acids, or specific to arginine.

**Figure 3:**
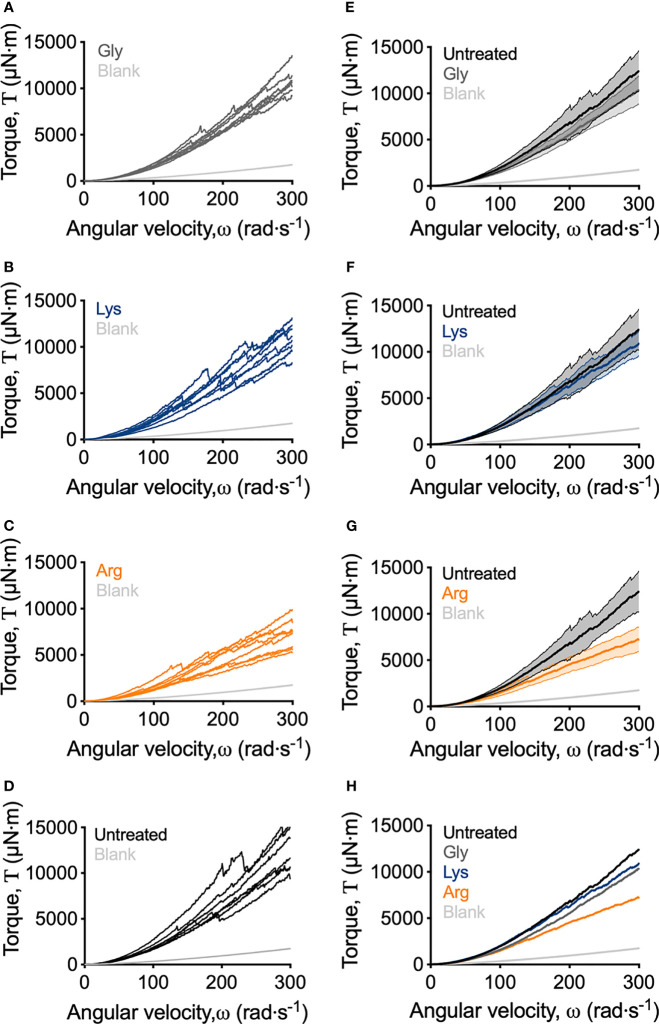
Adapted rotating-disc measurements of untreated and amino acid treated *S. gordonii* biofilms. **(A–D)** Torque – displacement curves of individual replicates of glycine-, lysine-, arginine-treated and untreated (labelled) *S. gordonii* biofilms. **(E–G)** Comparison of the torque – displacement profiles of glycine-, lysine- and arginine-treated biofilms to untreated biofilms (labelled). Data is presented as mean ± 95% confidence interval. **(H)** Data from **(A–D)** expressed as mean. Replicate graph with data presented as mean ± 95% confidence interval is depicted in **Figure S3**. In each panel, blank indicates analysis for coupon alone, with no biofilm. 4 biological replicates were performed, with 2 biofilms analyzed for each replicate (total N = 8).

**Figure 4:**
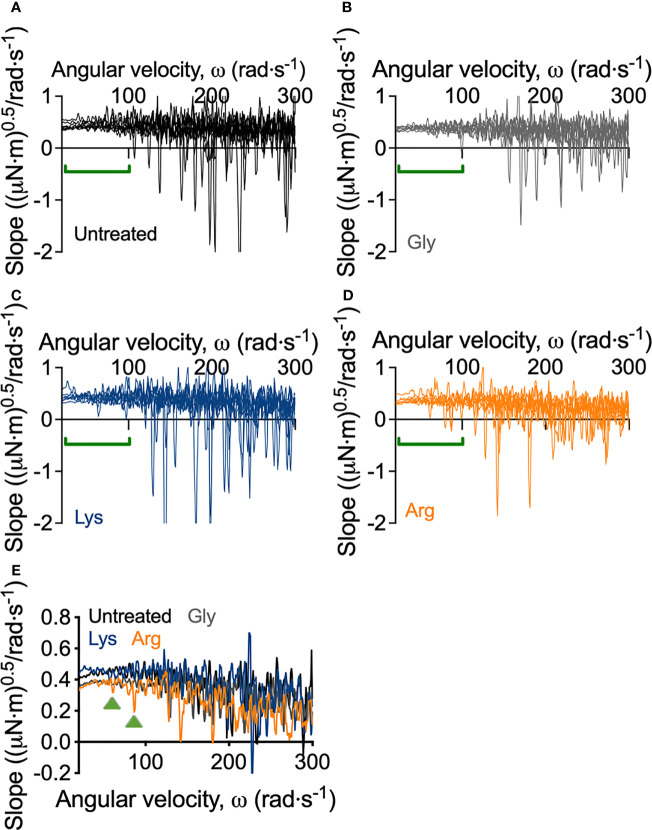
Transformed linearized analysis of untreated and amino acid treated *S. gordonii* biofilms. Curves of individual replicates of **(A)** untreated *S. gordonii* biofilms and biofilms treated with **(B)** glycine, **(C)** lysine, and **(D)** arginine. Data presented as mean ± 95% confidence interval is depicted in **Figure S4**. **(E)** Data from **(A–D)** presented as mean. Green brackets **(A–D)** and arrows **(E)** highlights regions where changes in torque, depicted here as negative slope values, were observed for arginine-treated biofilms, but not for glycine- or lysine-treated or untreated biofilms. 4 biological replicates were performed, with 2 biofilms analyzed for each replicate (total N = 8).

**Figure 5:**
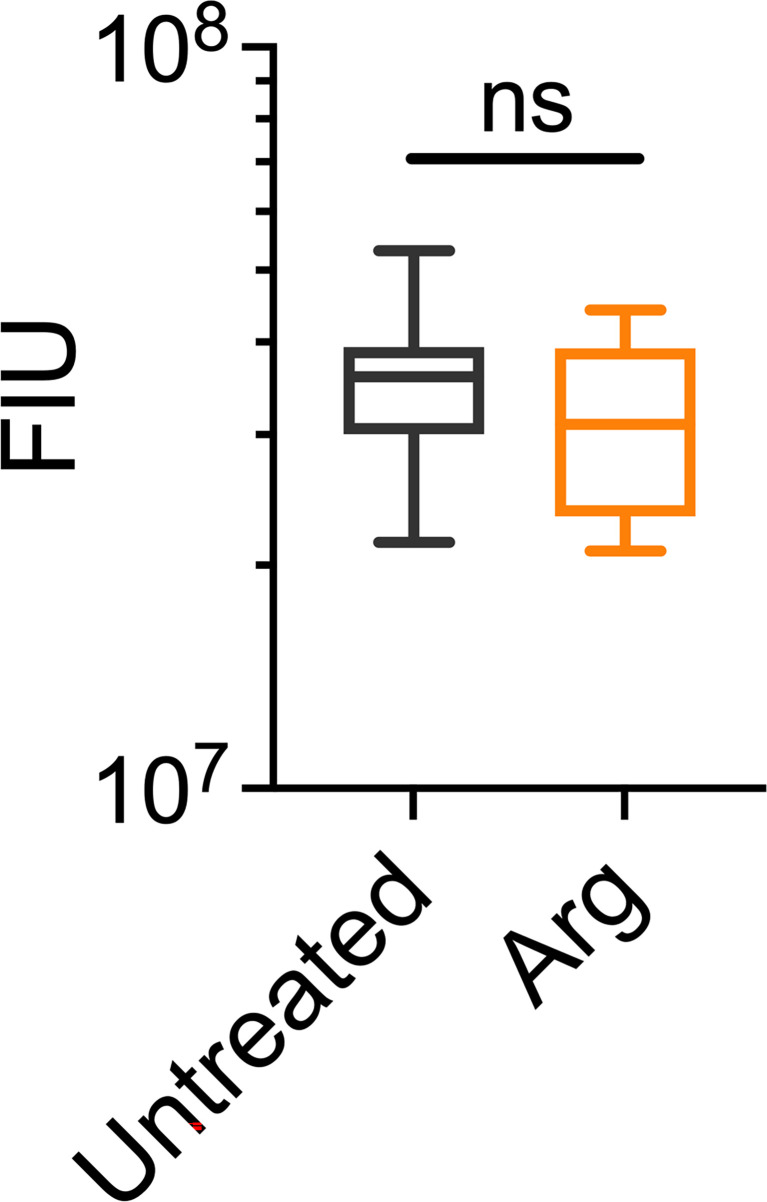
Arginine treatment does not lead to reduced biofilm biomass. 7 day *S. gordonii* biofilms were treated with either PBS (untreated control) or with 4% arginine for 2min. Biofilm biomass was removed from the coupon surface and labelled with Syto 9. Syto 9 signal is presented as fluorescence intensity units (FIU). N = 4; ns indicates no significant difference.

Macroscopically, arginine treatment did not appear to affect biofilm morphology, or the amount of remaining biofilm attached to the coupon after rheometry analysis (**Figures 1B, C**). However, arginine-treated biofilms displayed reduced torque, compared to untreated biofilms. In contrast glycine- and lysine-treated biofilms had similar torque – displacement profiles compared to untreated biofilms. These trends were true when considering the torque – displacement curves of individual biofilm replicates (**Figures 3A–D**) and combined data (**Figures 3E–H** and **Figure S3**). This indicates that coupons with arginine-treated biofilms could rotate more easily across the assayed angular velocity range. This is further highlighted by the transformed data (**Figure 4**) which amplified changes in torque that were occurring at lower angular velocity ranges that were not readily apparent in the torque – displacement curves (**Figure 3**). Visual inspection of this analysis revealed that changes in torque, indicated by negative slope values, were observed at lower angular velocity ranges for arginine-treated biofilms, compared to untreated and, glycine- and lysine-treated biofilms (**Figure 4**; green brackets and arrows, **Figure S4**). This suggests that biofilm detachment or rearrangement events were occurring at these lower angular velocity ranges for arginine-treated biofilms. Both treated and untreated biofilms had increased torque values compared to the coupon alone (**Figure 3**). The reduced torque of arginine-treated *S. gordonii* biofilms was not due to a reduction in biofilm biomass (**Figure 5**), suggesting that arginine treatment altered the mechanical properties of the biofilm.

**Figure 6:**
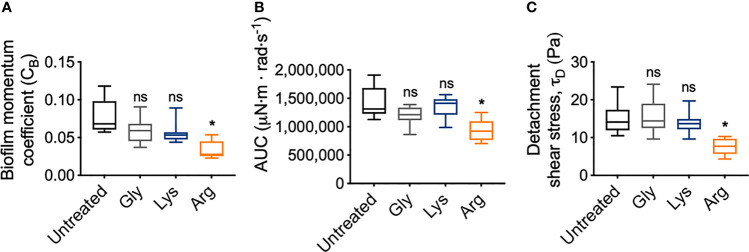
Arginine treatment weakens *S. gordonii* biofilms. **(A)** Biofilm momentum coefficient (*C_B_*), determined according to equation 1, at 200 – 300 rad.s^-1^ in **Figure 3** [Range; Untreated: 0.057 – 0.118; glycine: 0.037 – 0.091; lysine: 0.044 – 0.089; arginine: 0.023 – 0.054]. **(B)** Area under the curve (AUC) of torque – angular velocity curves depicted in **Figure 3** [Range; Untreated: 1,126,923 – 1,909,471; glycine: 863,187 – 1,390,189; lysine: 986,807 – 1,564,073; arginine: 702,523 – 1,250,791 μN·m · rad·s^-1^]. **(C)** Initiation of detachment, indicated as the first reduction in torque in **Figures 3**, converted to shear stress according to equ 3 [Range; Untreated: 10.50 – 23.50; glycine: 9.61 – 24.10; lysine: 9.61 – 19.70; arginine: 4.34 – 10.30 Pa]. * p-value < 0.05, ns indicates no statistical difference.

To quantify the mechanical differences between treated and untreated *S. gordonii* biofilms, the biofilm momentum coefficient across the turbulent regimes of 200 – 300 rad·s^-1^, was determined according the equation 1. The biofilm momentum coefficient is a dimensionless unit that is an indication of the drag caused by the biofilm, which in turn is related to the thickness and roughness of biofilm. Therefore, a higher coefficient is associated with more drag on the coupon, due to increased amount of adhered biofilm (Granville, 1982; Dennington et al., 2015). Glycine- and lysine-treated *S. gordonii* biofilms had biofilm momentum coefficients similar to untreated biofilms (**Figure 6A**). However, arginine-treated *S. gordonii* biofilms had a significantly lower biofilm momentum coefficient, compared to untreated biofilms (**Figure 6A**). This indicates that there was less drag caused by arginine-treated biofilms compared to either untreated or glycine- and lysine-treated biofilms.

To look into these differences further, the area under the curve (AUC) of the torque – angular velocity curves (**Figures 3A–D**) was determined (**Figure 6B**). Unlike the biofilm momentum coefficient, which only takes into consideration coupon rotation between 200 – 300 rad·s^-1^, AUC considers the rotation across the whole analyzed range. Consistent with the biofilm momentum coefficient analysis, there were no significant differences between the AUC of both untreated and glycine- and lysine-treated biofilms (**Figure 6B**). However, arginine-treated biofilms had significantly reduced AUC, compared to untreated biofilms (**Figure 6B**). This suggests that, when also considering the lower velocity ranges, less work was required for rotation of the coupon of arginine-treated *S. gordonii* biofilms, compared to both untreated biofilms and glycine- and lysine-treated biofilms.

As previously mentioned, visual inspection of the transformed analysis, suggested that for arginine-treated biofilms, reductions in torque, associated with biofilm detachment events, occurred at lower angular velocity ranges, compared to untreated biofilms (**Figure 4**; green brackets and arrows, **Figure S4**). However, interpretation of this transformed analysis is subjective. To therefore quantify these differences, the angular velocity where the first reduction in torque occurred was converted to the shear stress acting on the outer edge of the coupon, according to equ 3, providing an initiation of detachment shear stress quantification (**Figure 6C**). This analysis revealed that there was no significant difference in the detachment shear stress of glycine- or lysine-treated *S. gordonii* biofilms compared to untreated. However, reductions in torque occurred at significantly lower shear stresses for arginine-treated biofilms, compared to untreated (**Figure 6C**). This indicates that arginine-treated biofilms were detaching from coupons at lower shear stresses, suggesting that they were more easily removed by external shear forces, compared to untreated or glycine- and lysine-treated *S. gordonii* biofilms.

## Discussion

Arginine is emerging as a potential therapeutic to prevent oral diseases, due to its ability to maintain dental plaque-biofilm homeostasis and disrupt biofilm formation (Kolderman et al., 2015; Manus et al., 2018; Wolff and Schenkel, 2018). However, there remains little understanding of how arginine treatment impacts biofilm mechanics or detachment. Here we adapted rotating-disc rheometry from the field of biofouling (Granville, 1982; Dennington et al., 2015; Dennington et al., 2021), to study how shear induced removal of *S. gordonii* biofilms was affected by arginine treatment.

Our data suggest that *S. gordonii* biofilms appear to consist of two layers. An upper layer that was readily removed, and a base layer that was more adherent, and resistant to removal (**Figure S1**). This was true for both arginine-treated and untreated *S. gordonii* biofilms (**Figures 1B, C**). Similarly, a remaining biofilm layer that was resistant to removal when exposed to increasing shear stresses was observed for *S. mutans* biofilms (Hwang et al., 2014), and biofilms grown from river (Desmond et al., 2018) and drinking (Abe et al., 2012) waters (refer to **Supplementary Table 1** for a summary of biofilm growth and testing conditions). Mechanical heterogeneity across the biofilm *z-*plane architecture has also been quantified for *Pseudomonas fluorescens* (Cao et al., 2016) and *Escherichia coli* (Galy et al., 2012) biofilms using micro-rheology methods. Together, this suggests that a stratified mechanical architecture may occur in biofilms, resulting in a cohesion/adhesion gradient, with the base of the biofilm being rigid and highly resistant to external forces. This could have important implications when considering the mechanical and chemical removal of biofilms from surfaces.

Our analysis also revealed that arginine-treated *S. gordonii* biofilms had both reduced drag on the coupon during rotation (**Figures 6A, B**), and detached from the coupon at lower shear stresses (**Figure 6C**), compared to untreated biofilms. This suggests that arginine treatment weakened the structure of *S. gordonii* biofilms and that they were more easily removed from surfaces by external mechanical forces. Interestingly, previous observations of the biofilm disrupting effects of arginine either grew the biofilms in the presence of arginine, or treated the biofilms at multiple time points (Jakubovics et al., 2015; Kolderman et al., 2015; He et al., 2016). When mixed species biofilms were treated with arginine, three times a day over approximately 2 days, arginine effects to both microbial populations and biofilm structure were observed after 53 h (He et al., 2016). It was determined that arginine treatment takes time to exert effects on the biofilm, suggesting that arginine metabolism by arginolytic bacteria is required (He et al., 2016). However, here we observed arginine weakening *S. gordonii* biofilms after only 2 min of treatment. This suggests that mechanical destabilization of the biofilm can occur within a rapid time frame, compared to those that visually impact the biofilm architecture. These immediate mechanical effects are likely due to physical interactions, rather than metabolic. However, the biofilms analyzed here were thick (order of mm scale). As such there is the possibility that the exogenous arginine did not penetrate throughout the biofilm, particularly into the proposed rigid bottom biofilm layer, which was still attached to the coupon after analysis (**Figure 1C**).

AFM analysis of *S. mutans* biofilms, grown in the presence of arginine, identified that arginine reduced biofilm adhesion. *S. mutans* cannot metabolize arginine, and it was predicted that arginine prevented hydrogen bond interactions across glycan polymers within the EPS (Sharma et al., 2014). Furthermore, disruption of *S. gordonii* biofilms, when grown in the presence of high arginine concentrations, was predicted to be independent of arginine metabolism. Rather, it was predicted to be due to inhibition of cell-cell interactions within the biofilm (Jakubovics et al., 2015). We therefore predict that the weakening of arginine-treated *S. gordonii* biofilms observed here, may be due to disruption of chemical interactions between EPS components, or cell-cell or cell-EPS interactions within the biofilm. Similarly, *S. mutans* biofilms treated with a hydrolase that degrades EPS, were more easily removed from surfaces by exposure to external shear forces (Hwang et al., 2014). However, these biofilm destabilizing properties appear to be specific to arginine, and not a general action attributed to exogenous amino acids, as glycine or lysine treatment did not significantly alter *S. gordonii* biofilm mechanics compared to untreated biofilms (**Figures 3**, **6**).

Interestingly, *Pseudomonas aeruginosa* biofilms were more susceptible to tobramycin and ciprofloxacin treatment when the growth media was supplemented with arginine (Borriello et al., 2006). It was postulated that arginine was fermented in anoxic pockets of the mature biofilm, increasing the metabolic activity in these typically dormant regions and subsequently increasing the susceptibility to the antibiotic (Borriello et al., 2006). Our results suggest that arginine may also weaken the mechanical structure of the biofilm, allowing increased entry of the antibiotic into the biofilm. Together these results suggest that exogenous arginine can be used across multiple infection settings and has the potential to be used as an antimicrobial adjuvant.

Here we have adapted rotating-disc rheometry from the field of biofouling, as a novel methodology to analyze biofilm detachment from surfaces. We demonstrated that this assay is highly sensitive at detecting biofilm detachment, and possible structural rearrangements, with increasing shear forces. This methodology is also sensitive at detecting mechanical changes to the biofilm architecture that are not visually apparent. However, this method is destructive to the biofilm, and therefore, limits the sensitivity of assessing drag of the original structure at higher shears. Finally, we also identified, for the first time, that arginine treatment can weaken the mechanical structure of *S. gordonii* biofilms, resulting in detachment at lower shear stresses, compared to untreated biofilms. These effects were observed after only 2 min of treatment. Our results add to the multifaceted action of arginine at disrupting dental plaque-biofilms, and further promotes the potential use of arginine as an active compound in dentifrices to combat dental plaque and help improve oral health.

## Materials and Methods

### 3D Printing Coupons

The model for the coupons was designed in SolidWorks (Dassault Systèmes). The model is available through Dryad [https://doi.org/10.5061/dryad.jdfn2z3b2]. Coupons were 3D printed using a Prime 30 PolyJet 3D printer (Objet, Stratasys) using RGD720 photopolymer for the printing material (Stratasys). The coupon was printed at a resolution of 0.02 mm. The coupon surface was sanded used P300 sandpaper to create a rougher surface for bacteria to attach. Prior to inoculating, coupons were sterilized in 70% ethanol.

### *S. gordonii* Biofilm Growth and Treatment

*S. gordonii* wild type strain DL1 was used in this study. Overnight cultures were prepared by inoculating 10 mL of brain heart infusion broth (Oxoid; BHI) with a colony of *S. gordonii* and incubated statically overnight at 37°C with 5% CO_2_.

Sterile 40 mm coupons were placed in a Petri dish containing 40 mL BHI, supplemented with 0.5% sucrose. Coupons were inoculated with 400 μL of overnight culture. Biofilms were incubated in a humidified chamber at 37°C with 5% CO_2_, on an orbital shaker at 150 rpm. Every 24 h the media was replenished. Biofilms were grown for 7 days.

Biofilms were treated by transferring the coupons to a Petri dish containing either 40 mL PBS or 0.23M arginine, glycine, or lysine. This concentration was selected as it equated to 4% arginine, which has previously been shown to disrupt dental plaque-biofilms (Jakubovics et al., 2015). Amino acid solutions were normalized to pH 7. Biofilms were treated for 2 min at 37°C with 5% CO_2_, shaking at 150 rpm. Biofilms were washed in PBS and transferred to 40 mL PBS until analysis. 4 biological replicates were performed, each with duplicate biofilms.

### Adapted Rotating-Disc Rheometry Analysis

Biofilms were analyzed on a Discovery Hybrid Rheometer-2 (HD-2) (TA Instruments). A 15 x 15 cm square clear acrylic container filled with 2.8 L reverse osmosis water was transferred onto the Peltier plate. Biofilm-coated coupons were immersed and attached to the rheometer shaft using a custom-made adapter probe. The gap distance between the bottom of the container and the coupon was set to 3.5 cm (**Figure 1A**). Immersed coupons were spun at an angular velocity (ω) range of 0.1 – 300 rad·s^-1^, incrementing the speed across 360 s. It is important to note that the geometry of the system will influence the motion of water in the reservoir. As such measurements should be considered system-specific.

### Quantifying Biofilm Biomass

After treatment with either PBS (untreated control) or 4% arginine, *S. gordonii* biofilm biomass was scraped off the coupon using a cell scraper and resuspended in 5 mL PBS. Cellular aggregates were unable to be successfully disrupted by either sonication or syringe disruption. To therefore avoid these aggregates altering biomass quantification by colony forming units, biomass was quantified by labelling with Syto 9. Syto 9 is a green fluorescent membrane permeant nucleic acid stain, the signal of which increases when intercalated with nucleic acids. Therefore, Syto 9 will label all cells that contain DNA, and the presence of bacterial aggregates is predicted to have no impact on the fluorescent signal (Lebaron et al., 1998; Stocks, 2004). Styo 9 was diluted in PBS to a final concentration of 5 μM. 100 μL aliquots were transferred to the wells of a black 96-well plate. 100 μL aliquots of the treated or untreated biofilm suspension was added to the Styo 9 and incubated at room temperature for 15 min. Syto 9 fluorescence was measured on a SpectraMax i3 plate reader (Molecular Devices) as fluorescence intensity units (FIU) using an excitation of 485nm and emission of 535nm. 2 biological replicates, each with duplicate biofilms and four technical replicates were performed.

### Data Analysis

Data was collected using TRIOS v5 software (TA instruments), with raw data exported in excel. Data was transformed, and calculations performed in excel. Data was visualized and statistical analysis performed in GraphPad Prism v8 (GraphPad Software). All statistical comparisons were performed using a one-way ANOVA with a Tukey’s post-hoc test, with *p* < 0.05 indicating significance.

To more clearly observe the changes in torque, the torque – angular velocity curves were linearized and transformed (**Figure S2**). The data was linearized by taking the square root of the torque. The running slope of 5 data points of the linearized data was determined. This transformed data was linearized after 20 rad·s^-1^. Therefore, final transformed data is presented as the running slope of the linearized data against angular velocity, starting at 20 rad·s^-1^ (**Figure S2**). An example excel spreadsheet of the transformed data has been included in the supplemental data files.

The biofilm momentum coefficient (*C_B_*), also referred to as the momentum or torque coefficient, was determined as previously described (Dennington et al., 2015). The adapted rotating-disc rheology measurement is most sensitive at detecting changes in torque at the turbulent regime, between 200 – 300 rad·s^-1^. Torque within this range has a linear relationship to ω^2^, where the slope of this line (*T^1/^* ^2^ */ω*) equates to *C_B_* · *k*. Therefore, *C_B_* can be defined by equation 1:


(1) $$C_{B}=\frac{slope}{k}$$

where *k* is a constant for the system, defined by:


(2) $$k=\frac{\rho \cdot r^{5}}{2}$$

where ρ is the density of the fluid, in this case water (997 kg/m^3^) and r is the radius of the coupon (0.02 m).

The angular velocity where the first decrease in torque was detected was converted to the shear stress acting at the outer edge of the coupon (τ), as previously described (Hunsucker et al., 2016), according to equation 3:


(3) $$\tau =\sqrt{\tau_{\varphi }^{2}+\tau_{r}^{2}}$$

where, τ_φ_ is the shear stress acting in the circumferential direction and τ_r_ is the shear stress acting radially. This is intended as a system specific comparator, and not an absolute value that can be applied across other experimental designs or applications.

The shear stress acting in the circumferential direction is described by equation 4:


(4) τφ=ω2·r2(4.96·log10Re−5.74)2·ρ

where Re is the Reynolds number acting at the outer edge of the coupon described by equation 5:


(5) Re=ω·r2v

where v is the kinematic viscosity (9 x 10^-7^ m^2^·s^-1^).

The shear stress acting in the radial direction is described by equation 6:


(6) $$\tau_{r}=\alpha \cdot \tau_{\varphi }$$

where α is the skewness between the shear stress acting in both directions, and is described by equation 7:


(7) α=4.3954.96·log10Re−5.74)2−0.0107

Finally, the area under the curve (AUC) of the torque – angular velocity curves was determined using the analysis function in GraphPad Prism.

## Data Availability Statement

The raw data supporting the conclusions of this article will be made available by the authors, without undue reservation.

## Author Contributions

KW designed and printed the coupons. EG performed all experimental work. EG and PS analyzed and interpreted the experimental data. EG, DW, JM, CD, and PS wrote the manuscript. All authors gave their final approval and agree to be accountable for all aspects of the work.

## Funding

This work was funded by Colgate-Palmolive. EG was funded by an American Heart Association Career Development Award (19CDA34630005). DW and PS were funded by the National Institute of Health (DJW: R01AI134895 and R01AI143916) (PS: R01GM124436).

## Conflict of Interest

Authors CD and JM are employees of Colgate-Palmolive.

The remaining authors declare that the research was conducted in the absence of any commercial or financial relationships that could be construed as a potential conflict of interest.

The study received funding from Colgate-Palmolive. The funder had the following involvement with the study: study design, decision to publish and preparation of the manuscript.

## Publisher’s Note

All claims expressed in this article are solely those of the authors and do not necessarily represent those of their affiliated organizations, or those of the publisher, the editors and the reviewers. Any product that may be evaluated in this article, or claim that may be made by its manufacturer, is not guaranteed or endorsed by the publisher.
