# Supplementary material for: Arginine Induced Streptococcus gordonii Biofilm Detachment Using a Novel Rotating-Disc Rheometry Method
Source: Front Cell Infect Microbiol. 2021 Nov 5;11:784388. doi: 10.3389/fcimb.2021.784388 (PMC8602906; doi:10.3389/fcimb.2021.784388)
Supplement: Supplementary file 1 [file Presentation_1.pdf]

Arginine induced *Streptococcus gordonii* biofilm detachment using a novel rotating-disc rheometry method

Supplemental Information

Erin S. Gloag, Daniel J. Wozniak, Kevin L. Wolf, James G. Masters, Carlo Amorin Daep, Paul Stoodley

Supplemental Figures

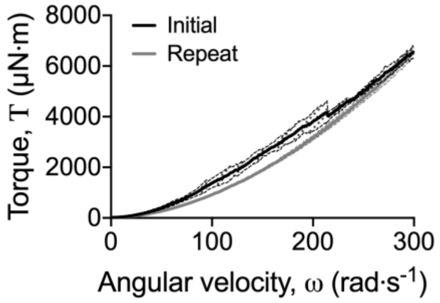

Supplemental Figure 1: Repeated analysis does not lead to further biofilm removal. Untreated *S. gordonii* biofilms were analyzed by adapted rotating-disc rheometry. After the initial measurement (black) the assay was repeated (grey) to determine if remaining attached biofilm could be removed with subsequent analysis. Repeated analysis revealed no changes in torque, and the curve reached the same final point as the initial analysis. This indicates that no additional biofilm removal was detected with repeated analysis. Data presented as mean ± SD, N = 4.

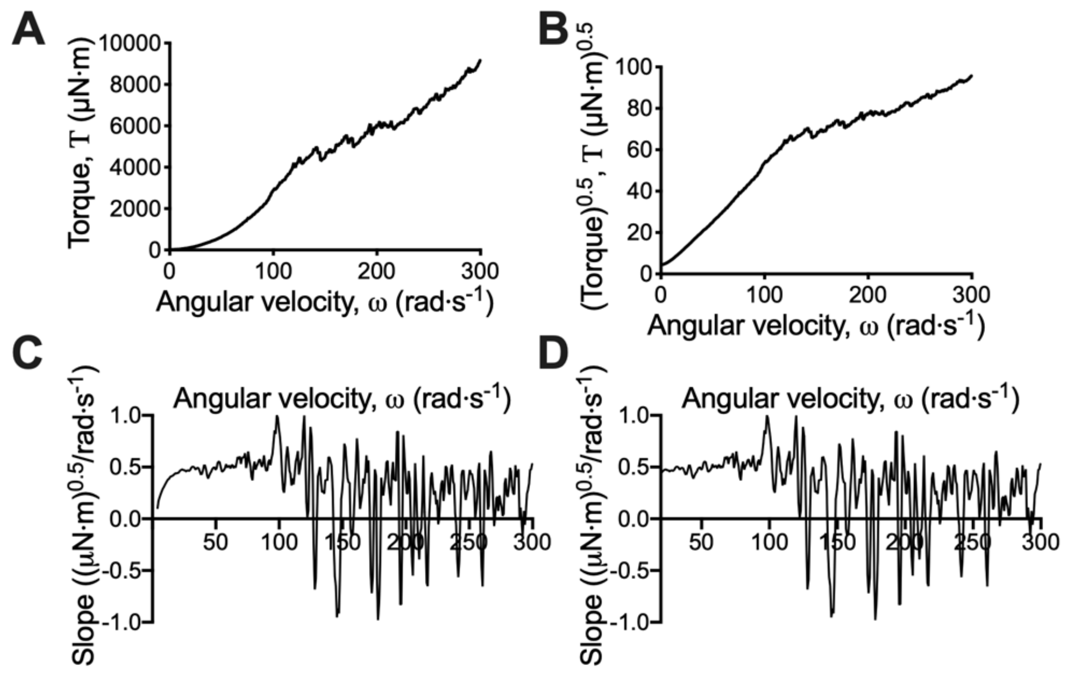

Supplementary Figure 2: Data processing. (A) The torque and angular velocity data were exported from TRIOS v5 software. To visualize the changes in torque with angular velocity more clearly the data was linearized by **(B)** plotting the square root of the torque against angular velocity. **(C)** The running slope of 5 consecutive data points of the linearized curve was determined. That is, the slope of data points 1 – 5, 2 – 6, 3 – 7 etc. was determined and plotted against the angular velocity. This analysis emphasized where changes in torque, which correlate to detachment events, were occurring which are now visualized as sharp peaks. **(D)** From this transformed data, the start of the curve had a sharp rise that was consistent across all data sets. To therefore focus on the linearized portion, data from 20 - 300 rad·s^{-1} was represented.

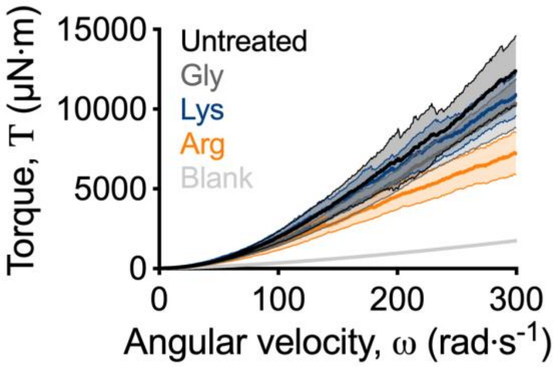

Supplemental Figure 3: Adapted rotating-disc measurements of untreated and amino acid treated *S. gordonii* biofilms. Torque – displacement curves of untreated and glycine-, lysine-, arginine-treated (labelled) 7 day *S. gordonii* biofilms. Data is presented as mean ± 95% confidence interval. 4 biological replicates were performed, with 2 biofilms analyzed for each replicate (total N = 8).

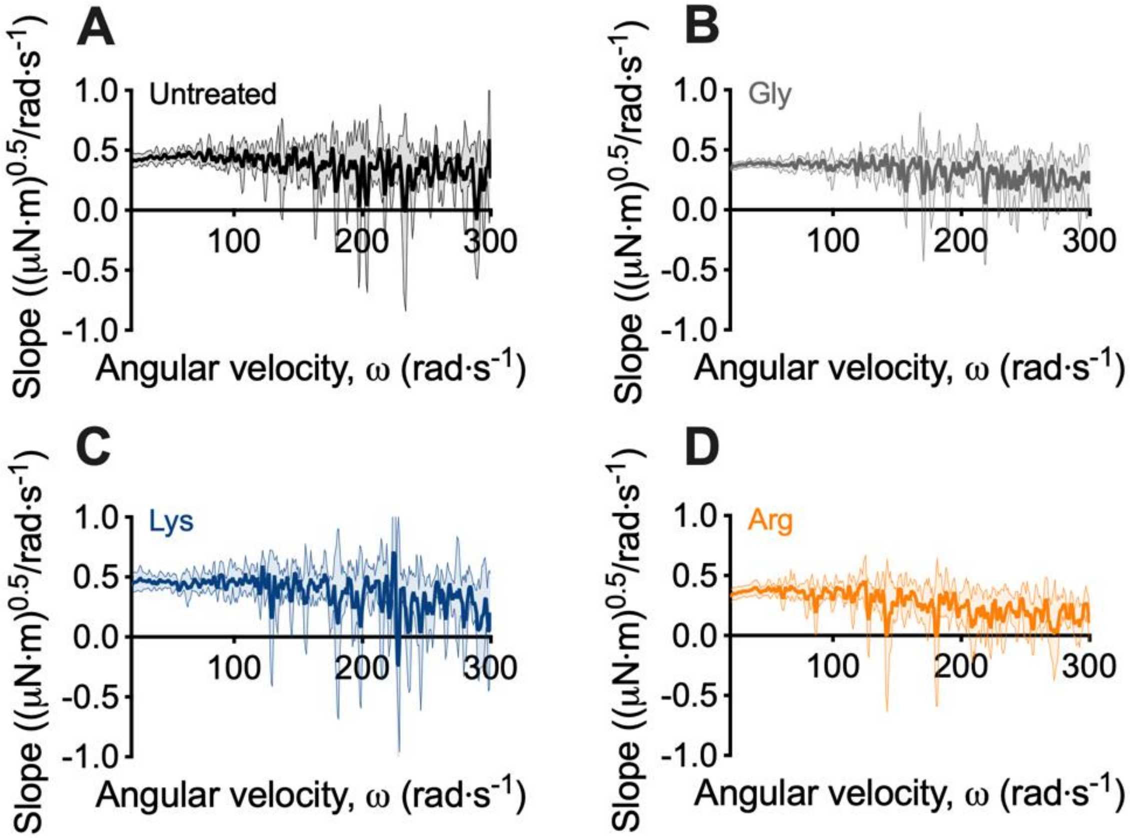

Supplementary Figure 4: Transformed linearized analysis of untreated and amino acid treated *S. gordonii* biofilms. Curves of **(A)** untreated *S. gordonii* biofilms and biofilms treated with **(B)** glycine, **(C)** lysine, and **(D)** arginine. Data are expressed as mean ± 95% confidence interval. 4 biological replicates were performed, with 2 biofilms analyzed for each replicate (total N = 8).

Supplementary Tables

Supplementary Table 1: Growth and testing conditions of biofilms with a remaining layer after exposure to shear stress

Inoculum	Substratum	Biofilm growth		Shear stress		Ref
		Model	Time	Method	Range	
S. mutans	Saliva-coated hydroxyapatite dics (12.7 mm diameter)	Static 24-well plate	67 or 115 h	Shear-induced biofilm mechanical strength tester (s-BMST) modelled on CDC reactor	Angular velocity flow of 0 – 115 rad×s^{-1} (estimated shear stress of 0 – 1.785 N m^{-2})	22
Untreated river water	Ultrafiltration membranes (18.75 cm^2 surface area)	Membrane fouling simulator	25 d	Peristaltic pump	Hydraulic shear stresses ranging from 0 – 2.6 Pa. Exposure time of 5 min at 0.2 Pa increments	23
Drinking tap water	Glass coupons (0.9 cm radius, 0.1 cm depth)	Rotating disc reactor	4, 8, 12 w	Atomic force microscopy	Set point range of -2 - 9 V which were the instrument limitations. This corresponded to a mechanical shear stress range of 5 - 300 kPa	24

Supplementary Movies

Supplemental Movie 1: Adapted rotating-disc rheometry measurement. Left panel is a recording of the rheometry measurement for an untreated 5 d *S. gordonii* biofilm. Right panel indicates the corresponding torque – angular velocity data collection. Individual frames from the time lapse depicting separate biofilm detachment events are displayed in Fig 2. Time stamp is indicated in the top left hand corner (min : s). Playback rate is at 15 fps. Movie S1 is available through Dryad [https://doi.org/10.5061/dryad.p8cz8w9q2].

Supplemental Movie 2: Transformed data collection. Left panel is the same recording depicted in movie S1. Right panel depicts the corresponding torque – angular velocity data that has been linearized and transformed to emphasis the changes in torque. Individual frames from the time lapse depicting separate biofilm detachment events are displayed in Fig 2. Time stamp is indicated in the top left hand corner (min : s). Playback rate is at 15 fps. Movie S2 is available through Dryad [https://doi.org/10.5061/dryad.p8cz8w9q2].
